# Hybrid coronary revascularization versus coronary artery bypass grafting for multivessel coronary artery disease: systematic review and meta-analysis

**DOI:** 10.1186/s13019-015-0262-5

**Published:** 2015-05-01

**Authors:** Peng Zhu, Pengyu Zhou, Yong Sun, Yilong Guo, Mingjie Mai, Shaoyi Zheng

**Affiliations:** 1 Department of Cardiovascular Surgery, Southern Medical University, Guangzhou, People's Republic of China; 2 Department of Cardiovascular Surgery, Guangdong General Hospital, Guangdong Academy of Medical Sciences, Guangzhou, People's Republic of China; 3 Department of Cardiovascular Surgery, Xiamen Heart Center, Xiamen, People's Republic of China

**Keywords:** Hybrid coronary revascularization, Coronary artery bypass grafting, Multivessel coronary artery disease, Meta-analysis

## Abstract

**Background:**

The concept of hybrid coronary revascularization (HCR) combines the left internal mammary artery (LIMA)–left anterior descending (LAD) graft and percutaneous coronary intervention (PCI) to non-LAD vessels. Multiple comparative studies have evaluated the safety and feasibility of HCR and coronary artery bypass grafting (CABG) for multivessel coronary artery disease (MCAD). However, the sample size of each study was small, and evidences based on single-institutional experience. The purpose of this meta-analysis was to compare the short-term outcomes of HCR with those of CABG for MCAD.

**Method:**

PubMed, EMBASE and Cochrane Library databases, as well as conference proceedings, were searched for eligible studies published up to March 2014. We calculated summary odds ratios (OR) for primary endpoints (death, stroke; myocardial infarction (MI); target vessel revascularization (TVR); major adverse cardiac or cerebrovascular events (MACCEs)) and secondary endpoints (atrial fibrillation (AF); renal failure; length of stay in the intensive care unit (LoS in ICU); length of stay in hospital (LoS in hospital); red blood cell (RBC) transfusion). Data from 6176 participants were derived from ten cohort studies.

**Results:**

HCR was non-inferior to CABG in terms of MACCEs during hospitalization (odds ratio (OR), 0.68, 95% confidence interval (CI), 0.34–1.33)and at one-year follow-up(0.32, 0.05–1.89) , and no significant difference was found between HCR and CABG groups in in-hospital and one-year follow-up outcomes of death, MI, stroke, the prevalence of AF and renal failure, whereas HCR was associated with a lower requirement of RBC transfusion and shorter LoS in ICU and LoS in hospital than CABG (weighted mean difference (WMD) –1.25, 95% CI, –1.62 to –0.88; –17.47, –31.01 to –3.93; –1.77, –3.07 to –0.46; respectively).

**Conclusion:**

Our meta-analysis indicates that HCR is feasible, safe and effective for the treatment of MCAD, with similar in-hospital and one-year follow-up outcome, significantly lower requirement of RBC transfusion, and faster recovery compared with CABG.

## Introduction

The revascularization strategy for multivessel coronary artery disease (MCAD) is associated with advantages and disadvantages. Coronary artery bypass grafting (CABG; on-pump and off-pump) offers superior long-term advantages owing largely to the left internal mammary artery (LIMA) to the left anterior descending (LAD) artery graft [1,2]. Conversely, CABG is a relatively aggressive surgical procedure with a higher risk of postoperative stroke [1,3-5], and conduits *via* the saphenous vein graft have comparatively short-term patency [6,7]. In contrast, percutaneous coronary intervention, a much less invasive method, carries a minimal procedural risk as well as a lower prevalence of failure for the target vessel due to the use of drug-eluting stents (DES) [8,9]. However, those benefits come at the expense of the need for repeat revascularization [2].

The concept of hybrid coronary revascularization (HCR), which combines the LIMA–LAD graft and percutaneous coronary intervention (PCI) to non-LAD vessels, was first introduced by Angelini and colleagues in 1996 [10]. Introduction of HCR has led to concerns as to whether HCR is superior to CABG for MCAD. Multiple comparative studies have evaluated the safety and feasibility of HCR and CABG for MCAD. However, the sample size of each study was small, and evidences based on single-institutional experience [11-21]. The purpose of this meta-analysis was to compare the short-term outcomes of HCR with those of CABG for MCAD.

## Review

### Materials and methods

#### Study selection and search strategy

Two independent reviewers (P.Z, and P.Y.Z) searched PubMed, Embase, Web of Science, and the Cochrane Library for randomized controlled trials (RCTs) and non-RCTs up to 1 March 2014 and compared HCR with CABG for MCAD without language or publication restrictions. The following medical subject heading terms and their variants were used in database searches: hybrid coronary revascularization; coronary artery bypass grafting (on-pump or off-pump); multivessel coronary artery disease. Reference lists within selected studies and abstracts published at major international conferences were also searched.

#### Outcome measures

The safety endpoints of this meta-analysis were death, stroke, myocardial infarction (MI) and major adverse cardiac or cerebrovascular events (MACCEs). The efficacy endpoint was revascularization. All the primary endpoints were measured in hospital and one year of follow-up. Death was defined as death from any cause. MI was diagnosed by symptoms, electrocardiography and changes in serum levels of cardiac biomarkers. Target vessel revascularization (TVR) was the need for repeated CABG or percutaneous coronary intervention (PCI). MACCEs were defined as a composite of death, MI, stroke or revascularization.

Secondary outcomes were atrial fibrillation (AF), renal failure (defined as an increase in serum creatinine values >25% above baseline values), length of stay in the intensive care unit (LoS in ICU), length of stay in hospital (LoS in hospital), and transfusion of red blood cells (RBCs).

#### Criteria for eligibility of inclusion of studies

Five main criteria were used: (i) comparison of HCR with CABG for MCAD; (ii) studies reporting at least one of the outcomes mentioned above; (iii) studies documenting surgical procedures such as one-stop HCR or staged HCR, on-pump or off-pump CABG, and documenting surgical methods such as HCR or CABG; (iv) follow-up duration ≥30 days; (v) non-RCTs with a score >5 as assessed by the Newcastle–Ottawa Scale (NOS) [22-24].

#### Assessment of the methodological quality of included studies

Three independent authors (Y.S., M.M.J, and Y.L.G) assessed the methodological quality of the included studies, and disagreement was resolved by consensus and discussion. Quality of non-RCTs was evaluated with the modified NOS (http://www.ohri.ca/programs/clinical_epidemiology/oxford.asp), which addressed three items: patient selection, comparability of groups, and outcome assessment (Table 1).

**Table 1:** **Newcastle-Ottawa scale used for methodological quality assessment of non-RCT**

Check list	
Selection	
1、	Assignment for treatment: any criteria reported? (if yes, 1 star)
2、	How representative was the reference group (CABG) in comparison with the general elderly population for CABG? (if yes,1 star; 0 star if the patients were selected or selection of group was not described)
3、	How representative was the treatment group (HCR) in comparison with the elderly population for CABG? (if drawn from the same community as the reference group, 1 star; 0 star if drawn from a different source or selection of group was not described)
Comparability	
4、	Group comparable for 1, 2, 3, 4,5 (if yes, 2 stars; one star was assigned if one of these five characteristics was not reported even if there were no other differences between the two groups and other characteristics had been controlled for; 0 star was assigned if the two groups differed)
5、	Group comparable for 6, 7, 8,9,10 (if yes, 2 stars; one star was assigned if one of these four characteristics was not reported even if there were no other differences between the two groups and other characteristics had been controlled for; 0 star was assigned if the two groups differed)
Outcome assessment	
6、	Clearly defined outcome of interest (yes, 1 star for information ascertained by record lincage or interview; 0 star if this information was not reported)
7、	Adequacy of follow-up (1 star if follow-up above 90%)

Comparability variables: 1 = age; 2 = gender; 3 = diabetes; 4 = hypertension; 5 = hypercholesterolemia; 6 = history of cerebrovascular disease; 7 = previous PCI; 8 = previous MI; 9 = smoking; 10 = PVD.

#### Statistical analyses

We undertook statistical analyses using Revman v5.2 (Cochrane Collaboration, Oxford, UK). Continuous and dichotomous variables were assessed by weighted mean differences (WMDs) and odds ratios (OR), respectively. A 95% confidence interval (CI) was recorded. Heterogeneity among studies was quantified using the I2 statistic. According to Higgins’ method, I^2^ < 25%, 25–50%, and >50 % were defined as low, moderate, and high heterogeneity, respectively [25]. A fixed-effect model was applied when I^2^ < 50%, and a random-effect model employed if I^2^ was >50%. *P* < 0.05 was considered significant. Sensitivity analyses were done on primary outcomes by changing the effects model and adjusting inclusion criteria. Publication bias was analyzed by funnel plots and evaluated by Egger’s test.

## Results

**Figure 1:**
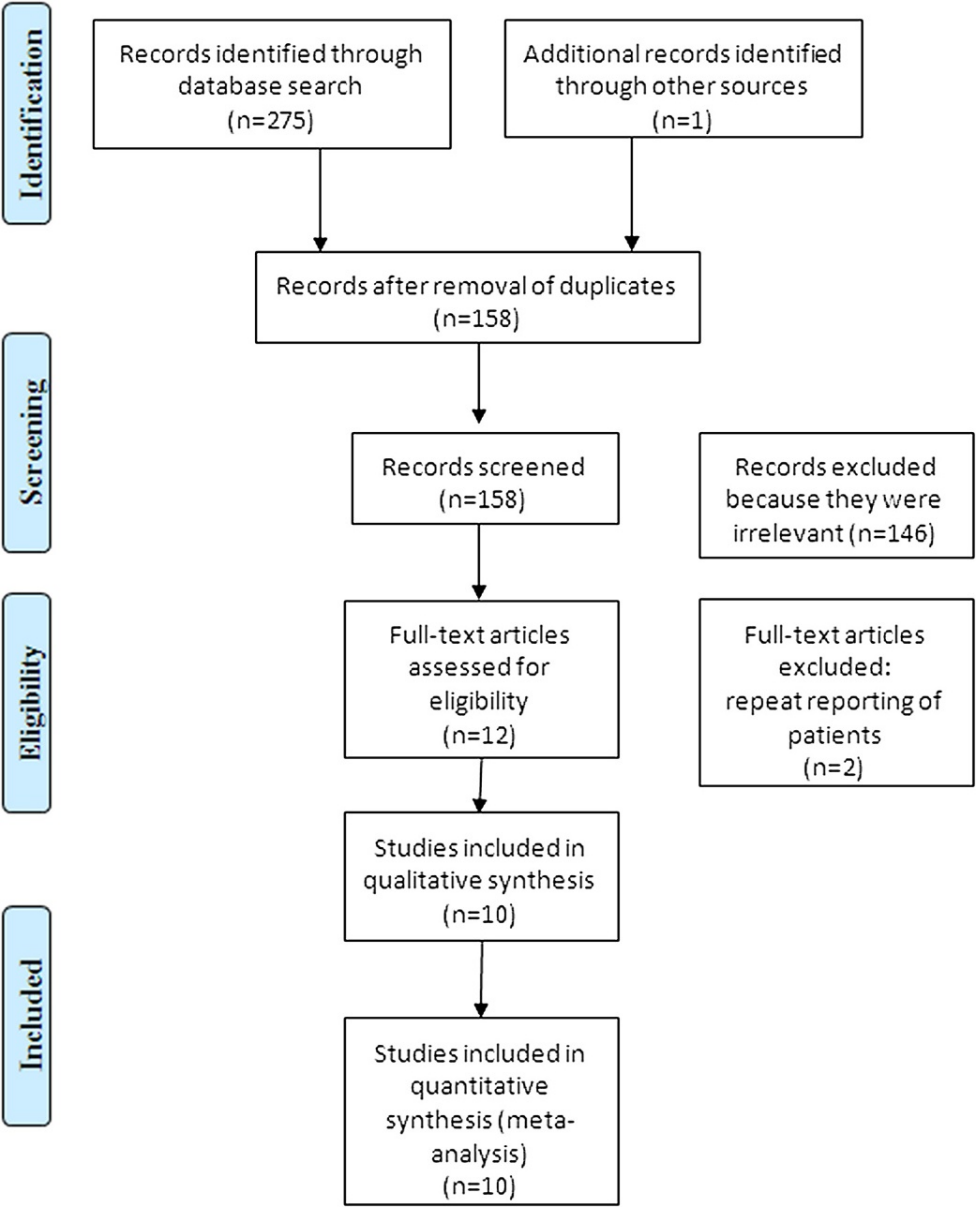
Flowchart of study identification and selection following PRISMA statement.

We identified ten studies eligible for inclusion: nine non-randomized and one randomized (Figure 1 and Table 2), All of the non-RCTs had a NOS score >5 [11,12,14,16-21], which was considered to denote a high-quality trial (Table 3). Figure 1 details the process of the identification and selection of studies following the PRISMA statement [26]. Baseline characteristics of patients in the Studies included is shown in Table 4. All studies combined represent outcome data on 6176 patients who underwent HCR(n = 623) or CABG surgery (n = 5553) from 2007 until present.

**Table 2:** **Main characteristics of included studies**

Study	Year	Numbers of patients (HCR/CABG)	Study design	CABG	HCR strategy	Follow-up (months)
Kon [17]	2007	15/30	Non-RCT	OPCABG	Simultaneous	12
Zhao [11]	2008	112/254	Non-RCT	On-pump	Simultaneous	NR
Reicher [14]	2007	13/26	Non-RCT	OPCABG	Simultaneous	14
Vassiliades [12]	2009	91/4175	Non-RCT	OPCABG	PCI then MICABG	12
					6.6%	
					MICABG then PCI	
					93.4%	
Delhaye [20]	2010	18/18	Non-RCT	On-pump	CABG then PCI	12
					OPCABG then PCI	
Hu [18]	2010	104/104	Non-RCT	OPCABG	Simultaneous	18 ± 7.9
Halkos [19]	2011	147/588	Non-RCT	OPCABG	Mainly staged	38.4 (median)
Bachinsky [21]	2012	25/27	Non-RCT	OPCABG	Simultaneous	1
Leacche [16]	2012	80/301	Non-RCT	OPCABG	NR	1
Popov [15]	Unpublished	18/30	RCT	On-pump	MICABG then PCI	1

Unless otherwise indicated, data are expressed as mean ± standard deviation.

PCI, percutaneous coronary intervention; OPCABG, on-pump coronary artery bypass grafting; MICABG, minimally invasive coronary artery bypass grafting; NR: not reported.

**Table 3:** **Assessment of quality of studies**

		Selection			Comparability		Outcome assessment		
Author	Year	1	2	3	4	5	6	7	Score
Kon	2007	*	*	*	*	**	*	-	*******
Zhao	2008	-	*	*	**	*	*	-	******
Reicher	2007	*	*	*	**	**	*	-	********
Vassiliades	2009	*	*	*	**	*	*	*	********
Delhaye	2010	*	*	*	**	*	*	-	*******
Hu	2010	*	*	*	**	**	*	*	*********
Halkos	2011	*	*	*	*	*	*	-	******
Bachinsky	2012	-	*	*	**	**	*	-	*******
Leacche	2012	-	*	*	**	*	*	-	******

-:zero point, *: One point, **: Two points.

**Table 4:** **Baseline characteristics of Patients in the Studies included**

Author	Group	N	Age (y)	Male (%)	Diabetes (%)	Hypertension (%)	Previous MI (%)	History of cerebrovascular disease (%)	Smoking (%)
Kon	HCR	15	61 ± 10	73	27	87	67	7	27
	CABG	30	65 ± 10	63	40	80	57	0	33
Zhao	HCR	112	63(32–85)	71	39	82	17	8	68
	CABG	254	63(32–89)	76	39	83	12	8	63
Reicher	HCR	13	62 ± 10	80	29	87	47	0	36
	CABG	26	64 ± 10	83	41	75	50	0	36
Vassiliades	HCR	91	65 ± 14	67	41	82	42	17	53
	CABG	4175	63 ± 12	69	37	83	48	18	69
Delhaye	HCR	18	62(55–70)	78	45	67	28	11	44
	CABG	18	60(53–68)	78	39	78	17	0	39
Hu	HCR	104	62 ± 10	11	25	60	34	9	56
	CABG	104	62 ± 8	20	27	63	29	6	37
Haloks	HCR	147	64 ± 13	62	60	87	17	20	42
	CABG	588	64 ± 13	71	64	85	12	17	50
Bachinsky	HCR	25	63 ± 11	80	36	72	20	4	28
	CABG	27	67 ± 11	59	48	96	44	15	22
Leacche	HCR	80	64	76	40	86	16	6	NR
	CABG	301	63	77	37	83	16	10	NR
Popov	HCR	18	60 ± 6	86	16	100	NR	14	NR
	CABG	30	59 ± 4	83	17	100	NR	0	NR

Data between parentheses represent median and 25th and 75th percentiles. Data with ± symbol represent mean and SD.

### Early outcomes (in hospital)

#### Primary clinical outcomes

##### Death

**Figure 2:**
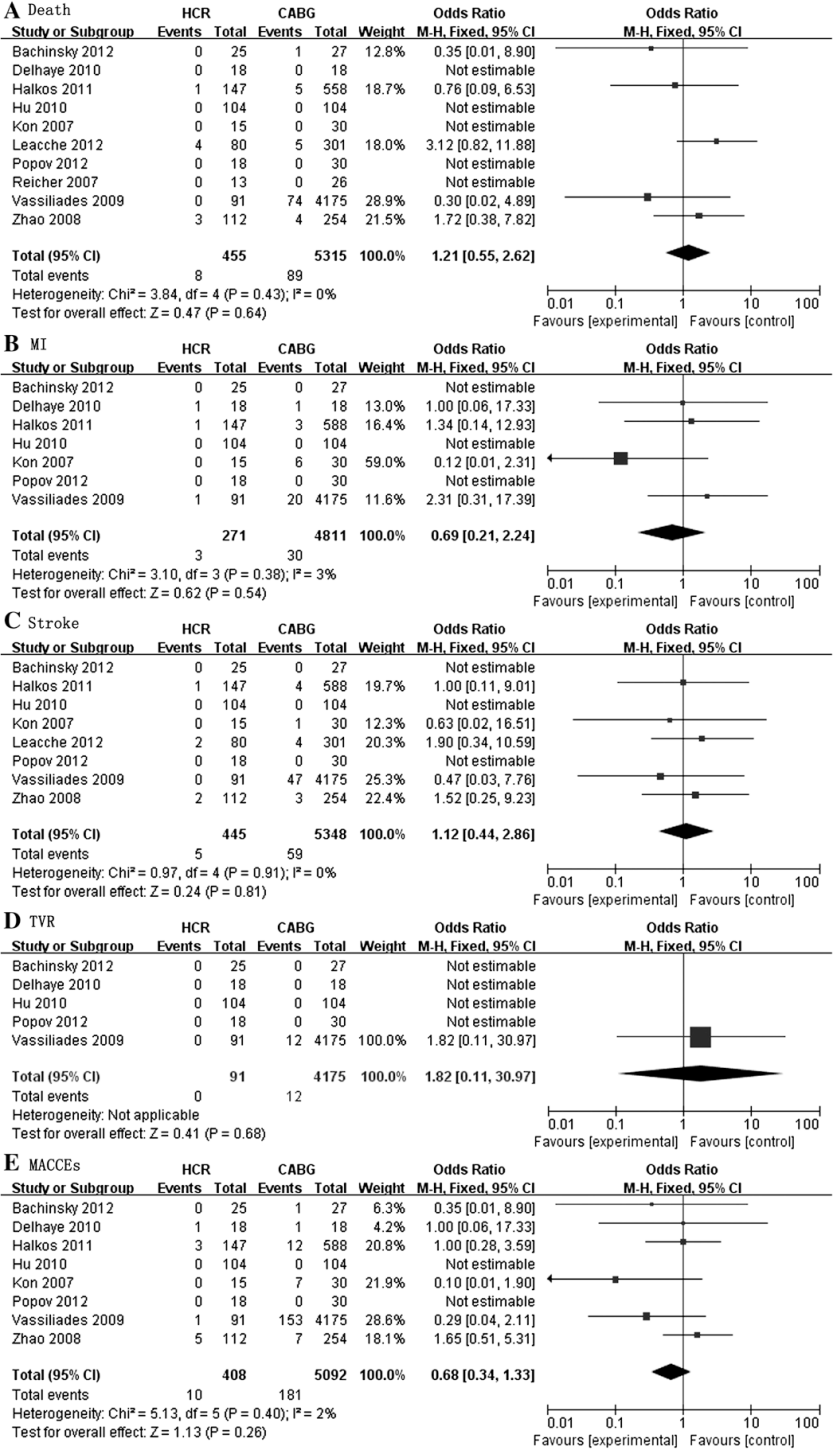
Forest plot showing a meta-analysis for HCR versus CABG during hospitalization. **A**. Death **B**. MI(Myocardial Infarction) **C**. Stroke **D**. MACCEs (Major Adverse Cardiac or Cerebrovascular Events).

Five studies reported on in-hospital mortality in 5770 patients. Pooled results showed no significant difference in mortality between the HCR group and CABG group <1 month (OR: 1.21; 95% CI: 0.55–2.62; *P* = 0.64; Figure 2A).

##### MI

Patients treated with HCR did not display a significant reduction in risk for MI as compared with those who received CABG within hospital (OR: 0.69; 95% CI: 0.21–2.24; *P* = 0.54; Figure 2B). Heterogeneity was not observed in this analysis (*I*^2^ = 3%).

##### Stroke

Stroke was assessed in five studies reporting on 5793 patients. The prevalence of stroke was not significantly different between groups. (OR: 1.12; 95% CI: 0.44–2.86; *P* = 0.81; Figure 2C). Heterogeneity was not observed in this analysis (*I*^2^ = 0%).

##### TVR

We analyzed the prevalence of TVR described in the five articles. For four studies, no event occurred in both groups, so we could not undertake a meta-analysis on the prevalence of TVR. In summary, there was insufficient evidence to show that the prevalence of TVR was different between HCR group and CABG group.

##### MACCEs

MACCEs occurred in 2.5% (10/408) of patients after HCR and 3.6% (181/5092) of patients with CABG.Six studies (5500 patients) provided data on the prevalence of MACCEs. Pooling of the outcomes of these studies revealed no significant differences in the prevalence of MACCEs between patients treated by HCR and those treated by CABG (OR: 0.68; 95% CI: 0.34–1.33; *P* = 0.26; Figure 2E). Slight heterogeneity was detected in this analysis (*I*^2^ = 2%).

### Secondary clinical outcomes

Five studies reported on AF. [11,16,18,19,21] Four studies reported renal failure [11,16,17,19,20]. Six studies reported LoS in ICU [14,15,17-19,21]. Five studies reported LoS in hospital [14,17-19,21]. Three studies reported transfusion of RBCs [14,17,21].

There was no significant difference in the prevalence of AF between the two groups (OR: 0.93; 95% CI: 0.70–1.23, *P* = 0.60) or the prevalence of renal failure (OR: 0.73; 95% CI: 0.36–1.49; *P* = 0.39).

HCR was associated with a significantly shorter LoS in ICU (29.99 *vs*.47.85 h; WMD: −17.47 h; 95% CI: −31.01 to −3.93; *P* = 0.01), LoS in hospital (5.44 *vs*. 7.30 days; WMD: −1.77 days; 95% CI, −3.07 to −0.46; *P* = 0.008), and fewer instances of RBC transfusion (0.26 *vs*. 1.55U; WMD: −1.25 U; 95% CI, −1.62 to −0.88; *P* < 0.001) (Table 5).

**Table 5:** **Results of meta-analysis of the secondary outcome**

Outcome measures	Number of studies	Patients (HCR/CABG)	I2 (%)	Analysis model	Statistics method	OR/WMD	95% CI	p value
AF	5	468/1274	0	Fixed	M-H	0.93	0.70,1.23	0.60
Renal Failure	5	372/1191	0	Fixed	M-H	0.64	0.32,1.27	0.20
Intubation Time	3	132/160	93	Random	IV	−9.95	−18.58,-1.31	0.02
LOS in ICU	6	322/805	85	Random	IV	−17.47	−31.01,-3.93	0.01
LOS in hospital	5	304/775	82	Random	IV	−1.77	−3.07,-0.46	0.008
Red Blood Cells Transfusion	3	53/83	0	Fixed	IV	−1.25	−1.62,-0.88	P<0.00001

OR odds ratio, WMD Weighted mean difference, M–H Mantel–Haenszel, IV inverse variance, CI confidence interval, AF atrial fibrillation, LOS in ICU lengths of stay in Intensive Care Unit, LOS in hospital lengths of stay in hospital.

### Longer-term outcomes (One year of follow-up)

**Figure 3:**
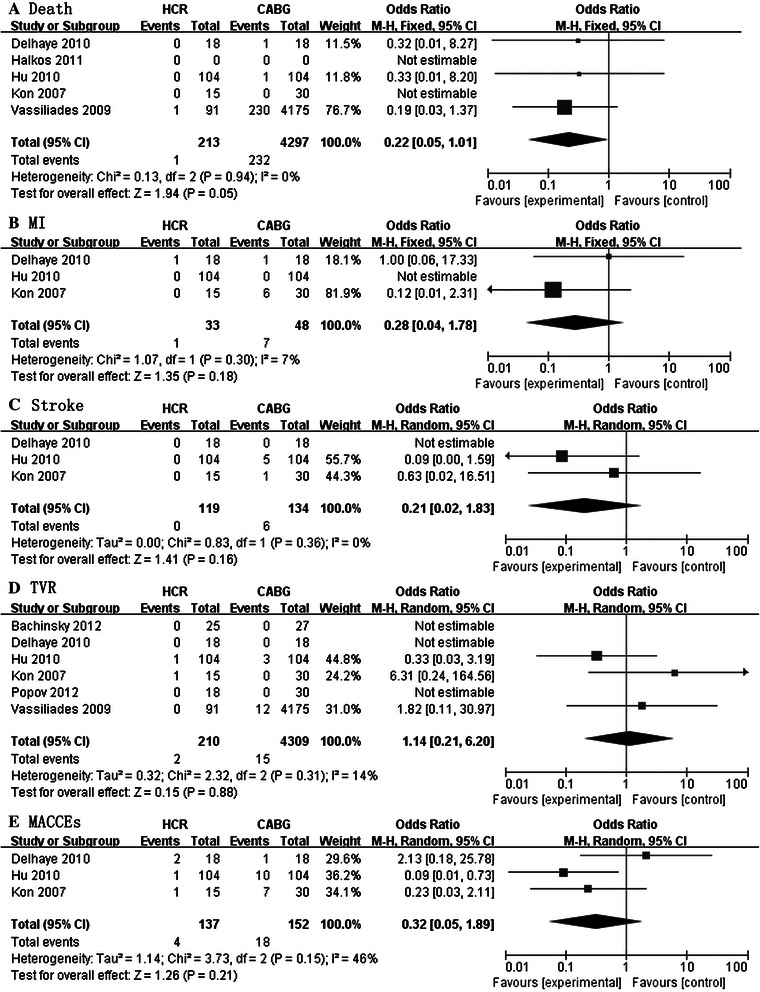
Forest plot showing a meta-analysis for HCR versus CABG at one-year of follow up. **A**. Death **B**. MI(Myocardial Infarction) **C**. Stroke **D**. TVR (Target Vessel Revascularization) E. MACCEs (Major Adverse Cardiac or Cerebrovascular Events).

MACCEs occurred in 2.9% (4/137) of patients after HCR and 11.8% (18/152) of patients with CABG at one year of follow-up. The ORs for MACCEs were not significantly different at one year of follow-up (OR: 0.32; 95% CI: 0.05–1.89, *P* = 0.21; Figure 3E ). As shown in Figure 3A, 3B, 3C, and 3D, overall, the outcomes for death, MI, Stroke and TVR at one year of follow-up were not statistically different.

### Sensitivity analyses and publication bias

Sensitivity analyses were undertaken on the outcomes mentioned above by re-analyses using a different effects model by including studies with ≥80 patients in each group and including non-RCTs with a score >6 as assessed by the NOS.

Analyses of non-RCTs studies with a score >6 separately also did not substantively alter the overall result of our analyses. Moreover, changing the model did not substantially change the pooled point estimate.

Inclusion of studies with ≥80 patients in each group did not substantially change the pooled point estimate except for LoS in ICU and LoS in hospital. Pooling the outcomes of these studies revealed no significant difference in the LoS in ICU (WMD: −10.47 h, 95% CI: −35.00 to 14.07; *P* = 0.40) or LoS in hospital (WMD: −0.42 days; 95% CI: −2.19 to 1.34; *P* = 0.64) between the two groups.

In summary, the results of sensitivity analyses supported the credibility of most of the evidences in this meta-analysis, but the credibility of the evidences about LoS in ICU and LoS in hospital should be considered carefully.

Also, we assessed for publication bias of data by Egger’s test and visual assessment of funnel plots. For the endpoint of in-hospital MACCEs, Egger’s test revealed *P* = 0.694, showing no evidence of publication bias.

## Discussion

In this meta-analysis, HCR was non-inferior to CABG in terms of in-hospital and one-year follow-up outcomes of death, MI, stroke, TVR, MACCEs, and some surgical complications (including AF and renal failure) whereas HCR was associated with a reduced need for RBC transfusion as well as shorter LoS in ICU and LoS in hospital than CABG.

We revealed no significant differences in the prevalence of in-hospital and one-year follow-up mortality, MI, stroke, TVR, MACCEs between the two groups, findings which were consistent with the early results of an ongoing RCT [15]. In that RCT, there was no mortality, MACCEs, or TVR in the hospital. The adequate design of that study (a prospective, randomized pilot trial to compare HCR with CABG in patients with MCAD) provided the preliminary data to strengthen the evidences of our study.

The prevalence of in-hospital one-year follow-up mortality, MI, stroke and MACCEs were similar between the two groups, however, we hypothesized that HCR may be superior to CABG in terms of long-term MACCEs. Hu et al. [18] reported a lower prevalence of MACCEs after HCR compared with on-pump CABG (1.0% *vs* 9.6%) after a mean follow-up of 18 months. Shen et al. [13] also reported that, after a mean follow-up of 3 years, in the high Euro-SCORE tertile, the prevalence of MACCEs in the hybrid group was significantly lower than that in the CABG group (*P* = 0.030).

As duration of follow-up extends, HCR may be superior to CABG in terms of long-term MACCEs.Several reasons support this findings mentioned above. Firstly, avoiding aortic clamping is one of the unique advantages of the hybrid procedure. Aortic manipulation (a predictor of postoperative cerebral infarction) is necessary during on-pump CABG [27]. Secondly, in the hybrid procedure, the quality of LIMA–LAD grafting is confirmed further by prompt angiography and deficiencies can be corrected immediately and reliably [28-30]. Finally, the less invasive nature of the hybrid procedure plays an important part in patient recovery.

Cardiac surgery and administration of contrast dyes during PCI tends to increase the risk of renal failure. Hence, we suggest that the prevalence of renal dysfunction might be higher in patients treated with HCR compared with those undergoing CABG alone. In this meta-analysis, however, no significant difference in the prevalence of renal failure was found between the two groups. The more stable hemodynamics and better urine output in the hybrid group may have resulted in a similar prevalence of renal failure in the present study [31,32].

In this analysis, the significantly lower requirement of RBC transfusion in the HCR group was attributed to the less invasive nature of minimally invasive direct coronary artery bypass grafting in the hybrid procedure despite continuous perioperative use of aspirin and perioperative administration of clopidogerl [12,14,31].

LoS in ICU and LoS in hospital were significantly shorter in the HCR group in this analysis, so the hybrid group had a shorter recovery with a substantial reduction in utilization of hospital resources [33]. This phenomenon could be explained in two ways. Firstly, a lower requirement of blood transfusion and reduced systemic inflammation have been associated with improved postoperative morbidity in a series of studies comparing minimally invasive and conventional surgery, and probably play a part in the outcomes of hybrid surgery. Secondly, better myocardial protection (as reflected by a reduction in regional release of myoglobin and systemic release of troponin I) might be another mechanism for quicker recovery after the hybrid procedure [17].

Heterogeneity among studies was observed for several continuous variables, including LoS in ICU and LoS in hospital. This heterogeneity may have resulted from variations in the surgeon’s caseload, the learning-curve effect, the HCR procedure, perioperative management, and standards regarding hospital discharge among the included studies.

### Limitations

This meta-analysis had several limitations. The main limitation of our meta-analysis was the retrospective nature of the available data. No RCT has been published but two protocols have been registered (available at http://www.clinicaltrials.gov/ct2/show/NCT01699048 and http://www.clinicaltrials.gov/ct2/show/NCT01035567). Ideally, a meta-analysis should include RCTs only, but inclusion of high-quality non-RCTs can improve the statistical power while maintaining an acceptable level of evidence. Abrahama et al. [34] reported that a meta-analysis of well-designed comparative non-RCTs of surgical procedures was as accurate as a meta-analysis of RCTs. Also, evaluation of publication bias cannot be done in a robust manner with such few data points, so the statistical power of Egger’s test to alert suspicion of publication bias was very limited in our meta-analysis. Therefore, more RCTs comparing HCR with CABG in patients with MCAD are necessary.

Secondly, the duration of clinical follow-up was limited to one year in most studies, whereas a meta-analysis of long-term outcomes was not possible due to insufficient data [12,13,17,18,20]. Hence, more long-term results will be necessary for future studies.

Thirdly, the definition of endpoints such as MI, MACCEs, and renal failure in different studies varied to some degree, which may have weakened the evidences in our analysis.

Finally, obvious heterogeneity was observed for several continuous variables. Therefore, the random-effects model was used.

## Conclusion

This meta-analysis suggested that HCR is feasible, safe and effective for treatment of MCAD, with similar in-hospital and one-year follow-up outcomes, significantly lower requirement for RBC transfusion, and faster recovery compared with CABG. It may provide a safe and effective alternative for treating selected patients with MCAD. However, to validate the long-term results of HCR for MCAD, more large-scale, multicenter, prospective RCTs are warranted.

## Abbreviations

HCR: Hybrid coronary revascularization
CABG: Coronary artery bypass grafting
MCAD: Multivessel coronary artery disease
OR: Odds ratios
MI: Myocardial infarction
TVR: Target vessel revascularization
MACCEs: Major adverse cardiac or cerebrovascular events
AF: Atrial fibrillation
LoS: Length of stay
ICU: Intensive care unit
RBC: Red bloodcell
CI: Confidence iinterval
WMD: Weighted mean difference
LIMA: Left internal mammary artery
LAD: Left anterior descending
DES: Drug-eluting stents
PCI: Percutaneous coronary intervention
RCTs: Randomized controlled trials
NOS: Newcastle–ottawa scale
